# Exosome-derived lncRNA A1BG-AS1 attenuates the progression of prostate cancer depending on ZC3H13-mediated m6A modification

**DOI:** 10.1186/s13008-024-00110-4

**Published:** 2024-02-13

**Authors:** Zhi Yang, Yu Luo, Fan Zhang, Likun Ma

**Affiliations:** Department of Urology, The Sixth Hospital of Wuhan, Affiliated Hospital of Jianghan University, No. 168, Hong Kong Road, Jiangan District, Wuhan, 430015 Hubei China

**Keywords:** Exosome, lncRNA A1BG-AS1, Prostate cancer, ZC3H13, m^6^A modification

## Abstract

**Background:**

Exosome-derived long non-coding RNAs (lncRNAs) and N6-methyladenosine (m^6^A) modifications of lncRNAs have been shown crucial functions in prostate cancer (PCa). Herein, we aim to investigate the detailed mechanism of exosome-derived lncRNA A1BG-AS1 in PCa process.

**Methods:**

PCa cell exosomes were extracted, exosomal marker proteins (CD63, CD9) were detected utilizing western blotting, and exosomes with overexpressing A1BG-AS1 were co-cultured with targeted PCa cells. qRT-PCR was used to detect A1BG-AS1 expression and m^6^A methyltransferase ZC3H13 in PCa. Transwell, colony formation and CCK-8 assays were utilized to assess the invasion, migration, and proliferation ability of PCa cells. Then, we performed actinomycin D and MeRIP assays to analyze the regulatory effect of ZC3H13 on A1BG-AS1 mRNA stability and m^6^A modification level.

**Results:**

We observed that A1BG-AS1 and ZC3H13 expression was restricted in PCa tumors. The invasion, proliferation and migratory capacities of PCa cells could be inhibited by up-regulating A1BG-AS1 or by co-culturing with exosomes that up-regulate A1BG-AS1. Additionally, ZC3H13 promoted stable A1BG-AS1 expression by regulating the m^6^A level of A1BG-AS1.

**Conclusion:**

Exosomal A1BG-AS1 was m^6^A-modified by the m^6^A methyltransferase ZC3H13 to stabilize expression and thus prevent PCa cell malignancy. These findings offer a possible target for clinical therapy of PCa.

## Background

Prostate cancer (PCa) is a common cancer in men, with new cases accounting for more than one-fifth of newly diagnosed cases of the most common tumors worldwide in 2020 [1]. Studies have shown that PCa patients diagnosed early have excellent 5-year survival of approximately 98% [1]. However, for PCa patients diagnosed in the middle to late stages, existing treatment options do not significantly improve survival prognosis [2, 3]. Furthermore, targeted therapy of cancer biomarkers and specific mutated genes can be used for targeted therapies of PCa [4]. Therefore, it is urgent investigate the biological mechanisms underlying the PCa development to improve the therapeutic efficacy and prognosis of PCa patients.

With the development of genome-wide molecular localization, the diversity of molecular changes during tumor progression has been revealed [5]. Long non-coding RNAs (lncRNAs) are greater than 200 nucleotides, constitute the majority of the transcriptome, and do not encode proteins [6]. Numerous research have revealed lncRNAs serve complex and precise biological functions as emerging regulators in a variety of diseases, including malignant progression of tumors [7, 8]. For example, lncRNA NXTAR is identified as a PCa antioncogene, and its absence increases enzalutamide resistance as well as androgen receptor expression [9]. Exogenous lncRNA MEG3 increased apoptosis and decreased migration, viability, proliferation, epithelial mesenchymal transition and invasiveness in PCa cells [10]. LncRNA A1BG antisense RNA 1 (A1BG-AS1) was downregulated in liver cancer and associated with prognostic features such as microvascular infiltration of cancerous tissues, advanced tumor staging characteristics, high tumor grade, and associated with poor clinical outcomes [11]. However, the changes in expression and the regulatory role of A1BG-AS1 in PCa are unknown.

Exosomes are nanoscale extracellular vesicles with lipid bilayers containing biomolecules such as DNA, RNA, lipids and proteins that are produced and released from cells [12]. Exosomes have been reported to participate in numerous disease processes and tumor-derived exosomes are connected to the regulation of cancer cell migration, invasion and pre-metastasis through paracrine subversion of the local and distant microenvironment [13, 14]. Exosomal lncRNA-p21 levels are higher in urine samples from PCa patients than those from Benign prostatic hyperplasia disease patients, and its expression helps to differentiate between PCa and benign disease [15]. LncRNA NEAT1 in PCa cells can be transferred via exosomes to human bone marrow-derived mesenchymal stem cells, which in turn promotes PCa bone metastasis [16]. Linc01213 is upregulated in exosomes of androgen-independent PCa cell lines, promoting androgen-dependent PCa cell proliferation and inhibiting cell cycle block [17]. Nevertheless, whether exosomal A1BG-AS1 is involved in PCa progression is unknown.

N6-methyladenosine (m^6^A) is a class of post-transcriptional modified forms of eukaryotic mRNAs consisting of methyltransferases, demethylases, and read proteins that dynamically regulate mRNA stability, decay, nucleus retention, and translational control [18, 19]. M^6^A participated in the regulation of PCa development. For example, methyltransferase METTL3-mediated m^6^A modification promotes up-regulation of lncRNA PCAT6 in an IGF2BP2-dependent manner, which in turn facilitates bone-metastatic PCa development [20]. LncRNA PVT1 expression in PCa is mediated to be up-regulated by m^6^A modification, which induces PCa cells to become more proliferative, migratory and invasive [21]. Zinc finger CCCH-type containing 13 (ZC3H13) is an important cancer-related RNA-binding protein, and has been found to be an m6A regulator that acts as a methylation transferase. However, the mechanism of ZC3H13 and A1BG-AS1 in PCa needs to be further investigated.

This study centered on the differential expression of A1BG-AS1 in PCa and its effect on the malignant behavior of PCa cells. In addition, we explored the molecular mechanisms by which exosomal A1BG-AS1 acts on PCa progression. This study will provide support for the notion that PCa-derived exosomal A1BG-AS1 can be used as a biomarker for PCa diagnosis.

## Results

### A1BG-AS1 is downregulated in PCa

**Fig. 1:**
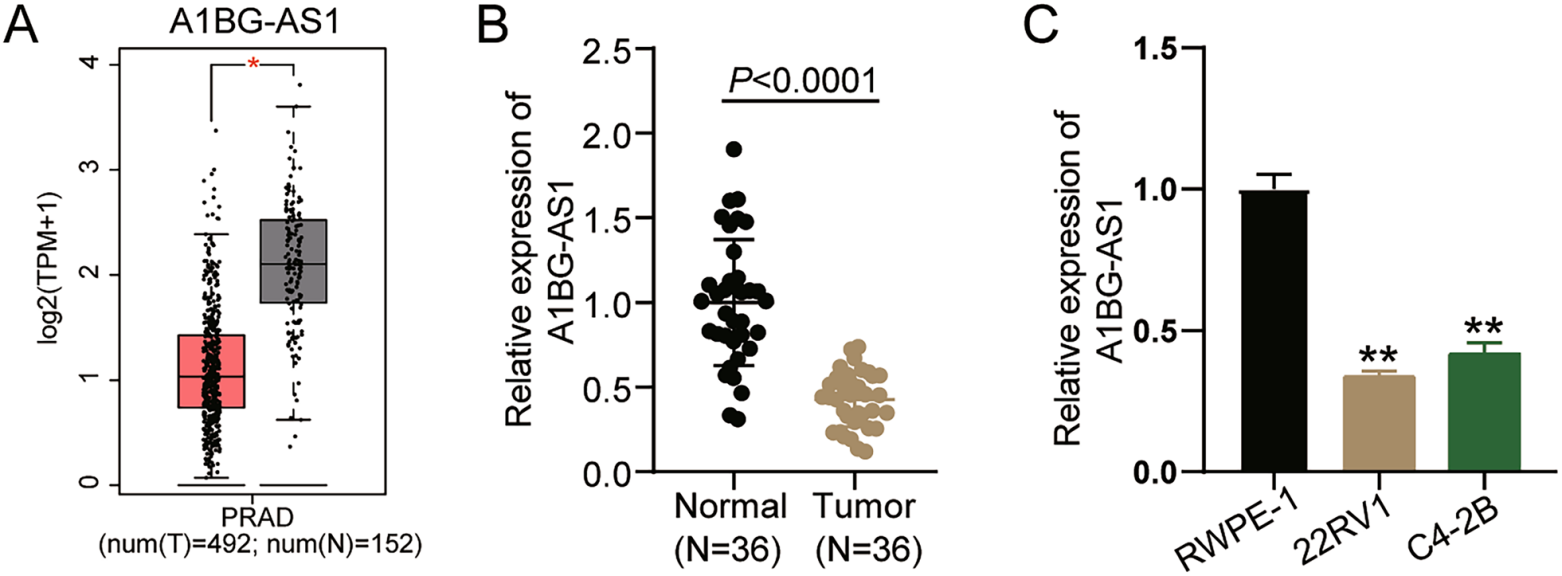
A1BG-AS1 is downregulated in PCa. **A** The level of A1BG-AS1 in PCa samples according to the data from GEPIA. **B** The levels of A1BG-AS1 in PCa and normal tissues were revealed via qRT-PCR. **D** The levels of A1BG-AS1 in PCa cells (22RV1 and C4-2B) and human normal prostate epithelial cells (RWPE-1) were revealed via qRT-PCR. **P < 0.001 vs. RWPE-1

GEPIA, an online tool, stores the RNA expression data of multiple human cancer and normal samples [22]. This study employed GEPIA to assess the differential expression of lncRNAs in PCa, revealing the downregulation of the lncRNA A1BG-AS1 in PCa samples (Fig. 1A). Then, the tumor and adjacent normal tissues from 36 PCa patients were collected to further confirm the A1BG-AS1 expression in PCa tissues by qRT-PCR. The results indicated a 60% downregulation of A1BG-AS1 levels in tumor tissues compared to normal tissues (Fig. 1B). Following the culture of two prostate cancer cell lines (22RV1 and C4-2B) and a human normal prostate epithelial cell line (RWPE-1), the qRT-PCR analysis revealed a downregulation of A1BG-AS1 levels by more than 50% in prostate cancer cells (Fig. 1C). Together with the data from GEIPIA, clinical samples and cell samples, we confirmed that A1BG-AS1 was downregulated in PCa.

### A1BG-AS1 overexpression suppresses PCa cell malignancy

**Fig. 2:**
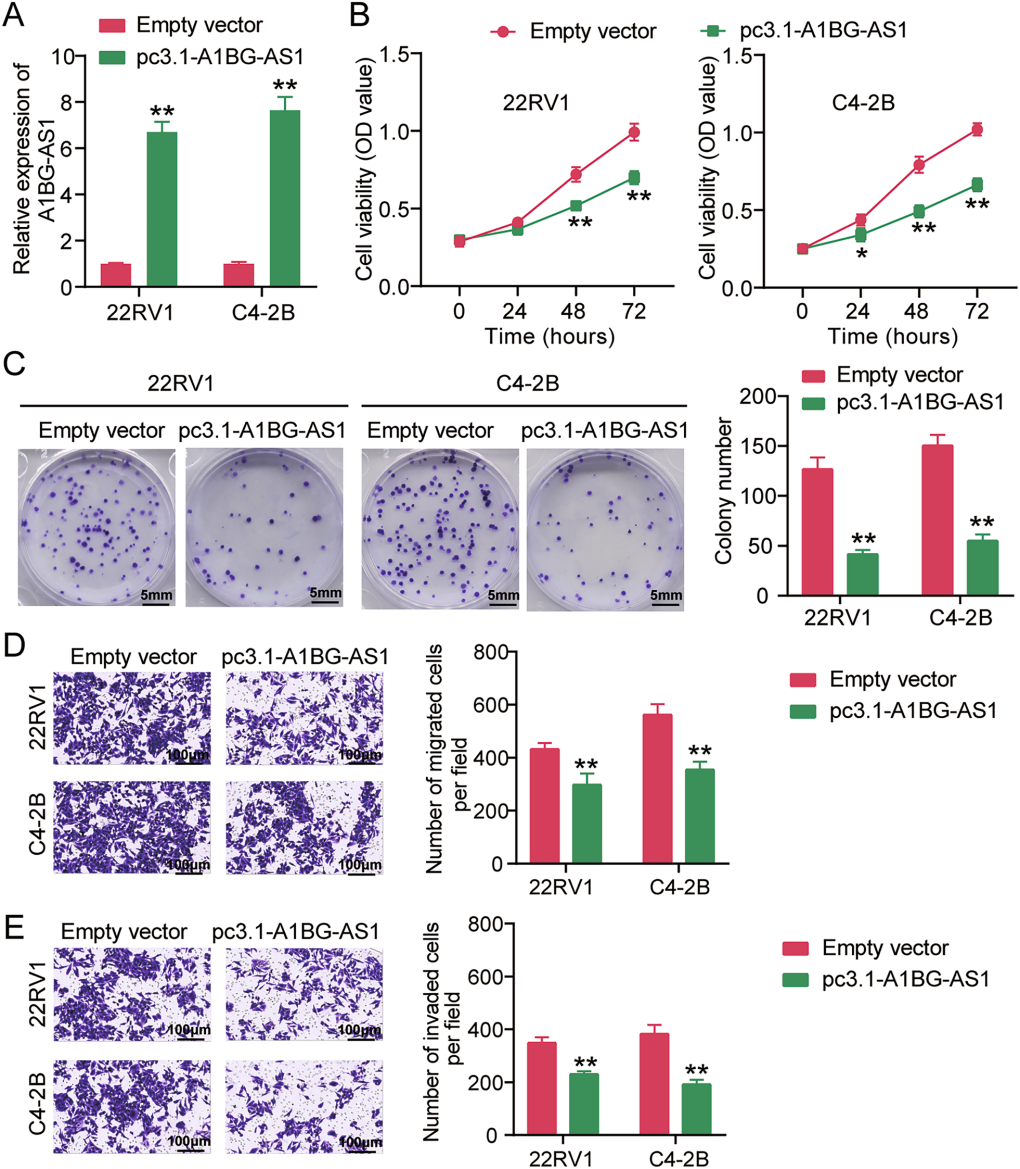
A1BG-AS1 overexpression suppresses PCa cell malignancy. **A** The levels of A1BG-AS1 in 22RV1 as well as C4-2B cells transfected with pc3.1-A1BG-AS1 or Empty vector were uncovered through qRT-PCR. **B**, **C** Cell proliferation of 22RV1 as well as C4-2B cells transfected with pc3.1-A1BG-AS1 or Empty vector was uncovered through CCK-8 assay (**B**) and colony formation assay (**C**). **D**, **E** Cell migration (**D**) and invasion (**E**) of 22RV1 as well as C4-2B cells transfected with pc3.1-A1BG-AS1 or Empty vector was uncovered through Transwell assay. **P < 0.001 vs. Empty vector

To confirm the effect of A1BG-AS1 on PCa cell malignancy, the A1BG-AS1 overexpression vector (pc3.1-A1BG-AS1) was transfected into PCa cells. qRT-PCR analysis, conducted to confirm transfection efficiency, revealed that pc3.1-A1BG-AS1 induced a more than sixfold upregulation of A1BG-AS1 in both 22RV1 and C4-2B cells, suggesting the high transfection efficiency of pc3.1-A1BG-AS1 in PCa cells (Fig. 2A). Then, CCK-8 assay was used to assess the effect of A1BG-AS1 overexpression on PCa cell proliferation. The results revealed that the OD value of PCa cells decreased after pc3.1-A1BG-AS1 transfection, indicating that A1BG-AS1 overexpression inhibited PCa cell proliferation (Fig. 2B). After performing colony formation assay to further confirm the effect of A1BG-AS1 overexpression on PCa cell proliferation, the colony number of PCa cells was reduced after A1BG-AS1 transfection, indicating that A1BG-AS1 overexpression inhibited PCa cell proliferation (Fig. 2C). After assessing the abilities of PCa cell migration and invasion by Transwell assay, A1BG-AS1 overexpression reduced the number of migrating and invading PCa cells, indicating that A1BG-AS1 overexpression impaired the abilities of PCa cell migration and invasion (Fig. 2D and E). Taken together, the above results proved that A1BG-AS1 overexpression suppressed the malignant phenotype of PCa cells.

### Exosome-derived A1BG-AS1 suppresses PCa cell malignancy

**Fig. 3:**
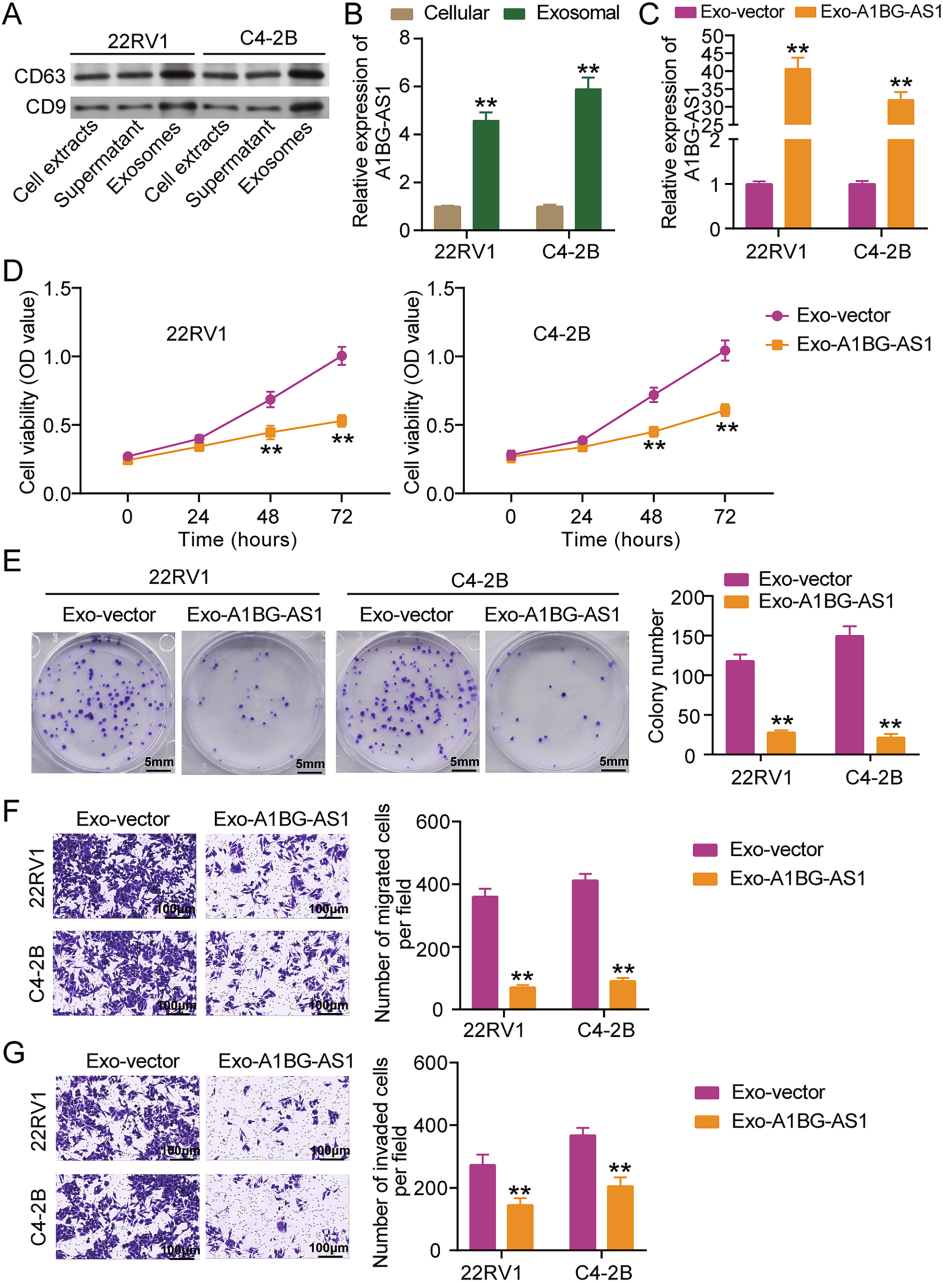
Exosome-derived A1BG-AS1 suppresses PCa cell malignancy. **A** The exosome markers (CD63 and CD9) were uncovered by western blot analysis. **B** The levels of A1BG-AS1 in cellular and Exosomal of 22RV1 as well as C4-2B cells were uncovered through qRT-PCR. **P < 0.001 vs. Cellular. **C** The levels of A1BG-AS1 in 22RV1 as well as C4-2B cells in Exo-vector and Exo-A1BG-AS1 groups were uncovered through qRT-PCR. **D** Cell viability of 22RV1 as well as C4-2B cells in Exo-vector and Exo-A1BG-AS1 groups were uncovered through CCK-8 assay. **E** Cell proliferation of 22RV1 as well as C4-2B cells in Exo-vector and Exo-A1BG-AS1 groups were uncovered through colony formation assay. **F** Cell migration of 22RV1 as well as C4-2B cells in Exo-vector and Exo-A1BG-AS1 groups were uncovered through Transwell assay. **G** Cell invasion of 22RV1 as well as C4-2B cells in Exo-vector and Exo-A1BG-AS1 groups were uncovered through Transwell assay. **C**–**G** Exo-vector group, the exosomes of 22RV1 cells and C4-2B cells in Empty vector group were co-cultured with 22RV1 cells and C4-2B cells, respectively. Exo-A1BG-AS1 group, the exosomes of 22RV1 cells and C4-2B cells in pc3.1-A1BG-AS1 group were co-cultured with 22RV1 cells and C4-2B cells, respectively. **P < 0.001 vs. Exo-vector

Recent studies have demonstrated that tumor cell-secreted exosomes containing lncRNAs can play a regulatory role in tumorigenesis [23–25]. Therefore, this study explored whether A1BG-AS1 was enriched in exosomes to regulate PCa cell malignancy. Isolation of exosomes from PCa cells was confirmed by western blot assay, which detected high expression of exosomal marker proteins (CD63 and CD9), verifying the successful extraction of exosomes from PCa cells (Fig. 3A). Then, qRT-PCR analysis detected A1BG-AS1 expression in exosomes, showing that A1BG-AS1 levels were upregulated by > 4.5-fold in exosomes (Fig. 3B). Furthermore, qRT-PCR analysis also found that A1BG-AS1 levels were more enriched in the exosomes from 22RV1 and C4-2B cells transfected with A1BG-AS1 overexpression vectors (Fig. 3C). To confirm the effect of exosome-derived A1BG-AS1 on PCa cell malignancy, exosomes from 22RV1 and C4-2B cells transfected with A1BG-AS1 overexpression vectors were co-cultured with PCa cells for a series of cell function experiments. The data from CCK8 and colony formation assays revealed that exosome-derived A1BG-AS1 suppressed PCa cell proliferation (Fig. 3D and E). Moreover, the transwell assay proved that the abilities of PCa cell migration and invasion were both impaired by exosome-derived A1BG-AS1 (Fig. 3F and G). Taken together, we concluded that exosome-derived A1BG-AS1 blocks the malignant phenotype of PCa cell.

### ZC3H13 mediates stable expression of A1BG-AS1 with m^6^A modification in PCa

**Fig. 4:**
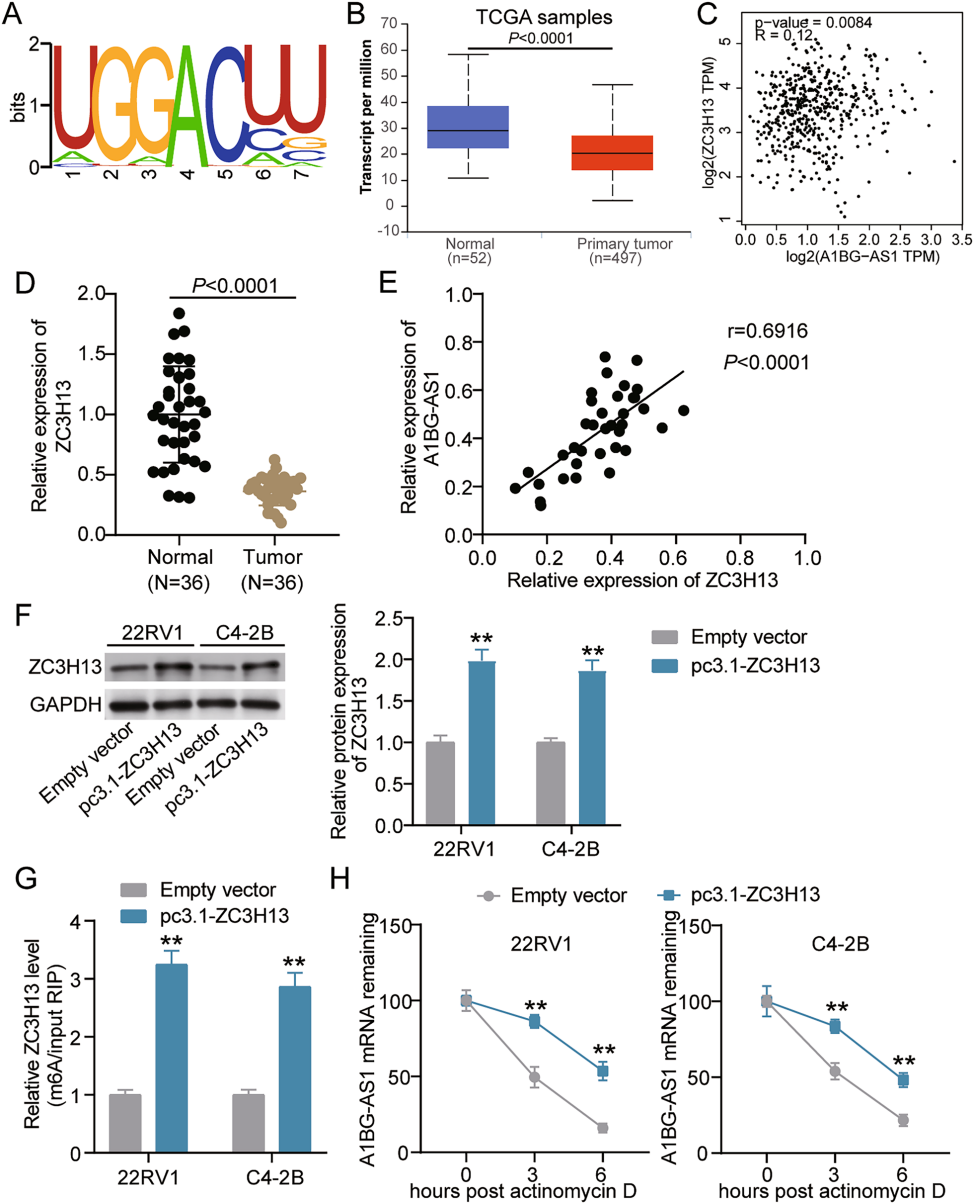
ZC3H13 mediates stable expression of A1BG-AS1 with m^6^A modification in PCa. **A** The m^6^A site of A1BG-AS1 was shown by RMBase v2.0. **B** The level of ZC3H13 in PCa samples were revealed by TCGA. **C** The correlation among A1BG-AS1 and ZC3H13 expression were revealed by TCGA. **D** The level of ZC3H13 in PCa samples normal tissues were revealed by qRT-PCR. **E** The correlation among A1BG-AS1 and ZC3H13 expression were revealed by Pearson analysis. **F** The level of ZC3H13 protein in 22RV1 as well as C4-2B cells treated with pc3.1-ZC3H13 or Empty vector were uncovered through western blotting. **G** The level of m^6^A modification of A1BG-AS1 was measured through MeRIP assay in 22RV1 as well as C4-2B cells treated with pc3.1-ZC3H13 or Empty vector. **H** The level of A1BG-AS1 in 22RV1 as well as C4-2B cells transfected with pc3.1-ZC3H13 or Empty vector after treated with actinomycin D at the indicated time points were detected by qRT-PCR. **P < 0.001 vs. Empty vector

The m^6^A modification of lncRNA has been demonstrated as a crucial mechanism in PCa progression [20, 26, 27]. This study deeply explored the m^6^A regulatory mechanism of A1BG-AS1 in PCa. RMBase v2.0 was firstly used to identify the m^6^A modification site of A1BG-AS1 (Fig. 4A). Then, according to the data from The Cancer Genome Atlas (TCGA), ZC3H13 was found to be downregulated in PCa samples, and ZC3H13 expression was positively correlated to A1BG-AS1 expression in PCa samples (Fig. 4B and C). Furthermore, qRT-PCR was performed to detect ZC3H13 expression in our collected tissues from 36 PCa patients. The results showed that ZC3H13 was downregulated by 64% in tumor tissues (Fig. 4D). Pearson correlation analysis results confirmed a positive correlation between ZC3H13 levels and A1BG-AS1 levels in PCa tissues (Fig. 4E). To confirm whether ZC3H13 could regulate A1BG-AS1 expression in PCa cells, the ZC3H13 overexpression vector (pc3.1-ZC3H13) was transfected into PCa cells. Western blot assay displayed that ZC3H13 protein levels in PCa cells increased more than 1.7-fold after pc3.1-ZC3H13 transfection, indicating the high transfection efficiency of pc3.1-ZC3H13 (Fig. 4F). Then, MeRIP assay was performed to confirm the m^6^A levels of A1BG-AS1 in PCa cells after pc3.1-ZC3H13 transfection. The results demonstrated that ZC3H13 overexpression induced approximately threefold increase of the m^6^A levels of A1BG-AS1 in PCa cells (Fig. 4G). Furthermore, after using actinomycin D to confirm the A1BG-AS1 mRNA stability in PCa cells transfected ZC3H13 overexpression vector, the A1BG-AS1 mRNA stability was enhanced in PCa cells with high-expressed ZC3H13 (Fig. 4H). All data mentioned above revealed that ZC3H13 increased the m^6^A level of A1BG-AS1 to enhance A1BG-AS1 expression in PCa cells.

## Discussion

Exosomes and the m6A modification have been confirmed as the key regulatory mechanism in PCa progression [20, 28–30]. Herein, A1BG-AS1 was low-expressed in PCa and its overexpression attenuated the tumorigenic behavior of cancer cells. Additionally, PCa cell-derived exosomes delivered A1BG-AS1 to target PCa cells and inhibited target PCa cell malignancy. Notably, we found that A1BG-AS1 was modified by ZC3H13 mediated m^6^A modification to promote stable expression. This provides new ideas for screening and treatment of PCa.

Accumulating reports reveal that A1BG-AS1 is aberrantly expressed in human cancers. Bai et al. [11] observed that A1BG-AS1 level was down-regulated in hepatocellular carcinoma, and that its enforced expression inhibited tumor cell proliferation, migration, and invasion in vitro, whereas the knockdown promoted the malignant behavior of cells. Cai et al. [31] found that A1BG-AS1 was high-expressed in breast cancer, and A1BG-AS1 removal accelerated cancer cell apoptosis and inhibited metastasis. Han et al*.* [32] confirmed that A1BG-AS1 was upregulated in osteosarcoma, and knocking down A1BG-AS1 impaired the malignancy of osteosarcoma cells. The previous studies demonstrate that the expression and role of A1BG-AS1 in different cancer types are different. In this study, we found for the first time that A1BG-AS1 was low-expressed in PCa, and its overexpression inhibited cancer cell malignancy, which was similar to the results of Bai et al. [11] in hepatocellular carcinoma, suggesting that A1BG-AS1 is a class of antioncogene in PCa.

Exosomes transport complex components such as carried nucleic acids, lipids, and proteins to reprogramme target cells to influence tumor progression and survival [14, 33]. The involvement of exosome-transported lncRNAs in cancer progression has long been reported. Zang et al. [34] observed that lncRNA UFC1 was high-expressed in tumor tissues and serum exosomes of lung cancer patients, and that exosome-derived lncRNA UFC1 inhibited the tumor cell cycle and promoted proliferation. Huang et al. [35] revealed that exosomes delivered loaded lncRNA MEG3 to target osteosarcoma cells, promoting the anti-osteosarcoma effect of lncRNA MEG3. Jiang et al. [36] reported that serum exosomal lncRNA HOXD-AS1 was up-regulated in patients with metastatic PCa, which was closely correlated with recurrence-free survival and progression-free survival of patients closely associated with it. In this study, we reveal for the first time that upregulated A1BG-AS1 is delivered to target PCa cells via exosomes, thereby inhibiting target PCa cell migration and proliferation. In addition, this study revealed that A1BG-AS1 was enriched in exosome derived from PCa cells, and exosome-derived A1BG-AS1 could inhibit PCa cell malignancy. Our study further provides the evidence supporting the mechanism by which exosomes facilitate intercellular communication through the transfer of proteins, lipids, RNA, and DNA, thereby regulating the progression of prostate cancer [37].

Accumulated investigation have found that ZC3H13 is lowly expressed in different tumors such as hepatocellular [38], thyroid [39], colorectal carcinoma [40], inhibit the occurrence and development of tumor. Furthermore, it was reported that ZC3H13 was down-regulated in PCa [41], which is consistent with the results of this study. Recently, ZC3H13 has been found to bind to RNAs with m^6^A sites and increasing the stability of these RNAs. For example, ZC3H13 mediates m^6^A modification of CENPK mRNA to increase translation of PHF10 in a YTHDF1-dependent manner, which in turn inhibits pancreatic cancer [42]. ZC3H13 overexpressed thyroid cancer cells have elevated m^6^A levels of IQGAP1 mRNA [39]. ZC3H13 regulates the DUOX1 gene through m^6^A methylation modification, thereby affecting iron death in laryngeal squamous cell carcinoma [43]. However, the regulatory effects of ZC3H13 on lncRNAs have not been reported in any disease. Herein, we found for the first time that ZC3H13 is involved in m^6^A modification in PCa. Moreover, ZC3H13 in PCa regulates the m^6^A modification of A1BG-AS1 to stabilize A1BG-AS1 expression. Currently, the role of m^6^A modification in lncRNA has been confirmed as a crucial regulatory mechanism in the tumorigenesis. For example, a lncRNA BLACAT3 underwent m^6^A modification mediated by ALKBH5, leading to the stabilization of BLACAT3 RNA structure and thereby promoting angiogenesis in bladder cancer [44]. Additionally, the methyltransferase METTL3 induced m6A modification of lncRNA NEAT1 to stabilize NEAT1 expression, thereby accelerating tumorigenesis in non-small cell lung cancer [45]. In this study, we propose a novel mechanism involving m^6^A modification of lncRNA in PCa. Specifically, we demonstrate that ZC3H13 mediates the m^6^A modification of A1BG-AS1, leading to the stabilization of A1BG-AS1 RNA structure and consequently inhibiting the development of PCa.

However, there are several areas in this study that need to be improved. Firstly, the influence of ZC3H13 on the biological effect of PCa cells should be clarified. Secondly, the metastatic effect of A1BG-AS1 to PCa in vivo needs to be investigated.

In all, our study demonstrated that A1BG-AS1 was markedly down-regulated in PCa, its overexpression inhibited PCa cell migration and proliferation, and that exosome-derived up-regulated A1BG-AS1 similarly inhibited cancer cell malignancy. In addition, ZC3H13 promote A1BG-AS1 expression in PCa may rely on m^6^A modification. This study provides a theoretical basis for the diagnosis of PCa exosomal A1BG-AS1.

## Methods

### Clinical sample

This study was approved by the Ethics Committee of The Sixth Hospital of Wuhan, Affiliated Hospital of Jianghan University. Thirty-six patients with pathologically confirmed PCa admitted to Wuhan Six Hospital and surgically removed the tumor and adjacent tissues were selected. The patients provided informed consent for the clinical study. The patient’s information is shown in Table 1.

**Table 1:** Clinical characteristics of prostate cancer patients in this study (N = 36)

Characteristics	No. of patients (%)
Age (years)	
≤ 60	16 (44.4)
> 60	20 (55.6)
Gleason score	
4–6	11 (30.6)
7	9 (25.0)
8–10	16 (44.4)
Focality	
Unifocal	13 (36.1)
Multifocal	23 (63.9)
T stage	
T1-T2	17 (47.2)
T3-T4	19 (52.8)

### Cell culture

All cell lines and culture medium were from BeNa Culture Collection (China). 22RV1 (Cat#: BNCC100161) and C4-2B (Cat#: BNCC341733) cells were PCa cells and cultured in RPMI-1640 medium containing 10% FBS and 1% P/S. RWPE-1 (Cat#: BNCC341583) cells were human normal prostate epithelial cells and cultured in KM medium containing 1% KGS and 1% P/S. All cell lines were maintained under and 37 ℃ with 5% CO_2_.

### Cell transfection

The day before transfection, 22RV1 and C4-2B cells were cultured in antibiotic-free growth medium to 50% confluence. After that, A1BG-AS1 or ZC3H13 high-expression pcDNA3.1 vectors (pc3.1-A1BG-AS1, pc3.1-ZC3H13) and Empty vector obtained from RiboBio (China) were transfected with Lipo6000 (Beyotime, China). After transfected cells were cultured for 48 h, RNA levels were measured to verify the transfection efficiency and to continue the relevant experiments.

### qRT-PCR

RNA Extraction Kit (Generay Biotech, China) was performed to extract total RNA, and HiScript 1st Strand cDNA Synthesis Kit (Vazyme, China) was used for subsequent cDNA synthesis experiments. PCR was performed on a Q3200 Real-Time PCR System (Bio-gener, China) with help of SYBR^®^ Green Master Mix (Takara, Japan). 2^−ΔΔCt^ was used to calculate the A1BG-AS1 and ZC3H13 expression using GAPDH as internal reference. The primers are shown in Table 2.

**Table 2:** PCR primers used in this study

Gene	Primer type	Sequence
A1BG-AS1	Forward	5ʹ-TTTAGTAGAGACGGGGTTTCGTC-3′
	Reverse	5ʹ-CTGATGGTTGCAAAGGAGTTTG-3′
ZC3H13	Forward	5ʹ-TCTGATAGCACATCCCGAAGA-3′
	Reverse	5ʹ-CAGCCAGTTACGGCACTGT-3′
GAPDH	Forward	5ʹ- CTGGGCTACACTGAGCACC-3′
	Reverse	5ʹ-AAGTGGTCGTTGAGGGCAATG-3′

### Western blot

Primary antibodies were CD63 (abs159125, Absin, China), CD9 (abs156046, Absin), ZC3H13 (abs134925, Absin) and GAPDH (abs132004, Absin). Secondary antibody was HRP-labelled Goat Anti-Rabbit IgG (A0208, Beyotime, China). Total proteins were extracted from 22RV1 and C4-2B cells via RIPA (Beyotime) and determined via BCA kit (Beyotime), then, electrophoresed on SDS-PAGE (10%) and transferred to a PVDF membrane, blocked and incubated with primary antibody at 4 ℃ overnight. After washing the membrane, it was incubated with secondary antibody for 1 h at 37 ℃. BeyoECL Plus (P0018S, Beyotime) was applied to observe the protein bands and protein levels were assessed using ImageJ software.

### Extraction, identification of exosomes and co-culture with PCa cells

pc3.1-A1BG-AS1 and Empty vector were transfected into 22RV1 and C4-2B cells, and cultured for 48 h. Then, centrifugation was performed at 4 ℃ at speeds of 1000 × *g* (10 min), 3000 × *g* (30 min) and 10,000 × *g* (60 min). After each centrifugation, the supernatant was filtered with 0.22 μm PVDF membrane to remove dead cells, cell fragments and displaced vesicles. Finally, the supernatant was centrifuged at 100000 × *g* for 4 h and exosome precipitates were harvested. The CD63 and CD81 levels on the surface of exosomes were determined by Western blotting. 30 μg exosomes were placed on 12-well plates for each group and 1 × 10^6^ 22RV1 and C4-2B cells were added, respectively, after 48 h of culture.

### CCK-8 assay

Transfected cells were seeded into 96-well plates with 2 × 10^3^ 22RV1 and C4-2B cells per well. At 0, 24, 48, and 72 h after cell inoculation, CCK-8 solution (Beyotime) was added and mixed gently. The cells were continued to culture for 2 h at 37 ℃ in an incubator. Optical density (OD) value was measured at 450 nm using a microplate reader (Molecular Devices, China).

### Colony formation assay

22RV1 and C4-2B cells (1 × 10^3^ cells/well) were injected into 6-well plates and cultured in complete medium for 14 days. After removing the culture medium and fixing the colonies for 15 min at 25 ℃ with 4% paraformaldehyde, the cells were then stained for 20 min with 0.1% crystal violet. Images were captured through Olympus light microscope (Japan).

### Transwell assay

In the migration assay, 1 × 10^4^ 22RV1 and C4-2B cells were suspended in serum-free medium and injected into the upper chamber of the Transwell with 100 μl, and the lower chamber of the Transwell was injected with 600 μl of medium containing 20% serum. After 24 h incubation at 37 ℃, the cells in the lower chamber of the Transwell were fixed as well as stained via crystal violet. The migrated cells were observed under the microscope. Additionally, the only difference in the invasion assay was that the upper chamber of the Transwell was pre-coated with Matrigel (BioFroxx, Germany).

### MeRIP-qPCR

m6A MeRIP Kit (GS-ET-001, Cloud-seq Biotech., China) was utilized to MeRIP assay. 22RV1 as well as C4-2B cells (1 × 10^7^) were lysed with lysis buffer, then, protein A/G beads (20 µl) conjugated with anti-m^6^A antibody (ab286164, Abcam, UK) or anti-IgG (ab133470, Abcam) were added and incubated overnight at 4 ℃. After extraction of RNA, A1BG-AS1 m^6^A-methylated level were detected through qRT-PCR.

### Actinomycin D assay

The 22RV1 as well as C4-2B cells in pc3.1-ZC3H13 and Empty vector group were exposed to 2 µg/ml of the transcriptional inhibitor actinomycin D for 0, 3, and 6 h to block transcription. Then, RNA was extracted, A1BG-AS1 levels were detected by qRT-PCR.

### Statistical analysis

Data from three replications are presented as mean ± SD, and analyzed via GraphPad Prism 8.0 software. The correlation among A1BG-AS1 and ZC3H13 was analyzed by Pearson. P < 0.05, difference statistically significant. The differences between multiple or two groups were analyzed via One way ANOVA or Student’s t-test, respectively.

## Data Availability

All data that had been produced and/or analyzed in the duration of this research have been appended in this manuscript.

## Abbreviations

PCa: Prostate cancer
m^6^A: N6-methyladenosine
lncRNA: Long non-coding RNA
A1BG-AS1: A1BG antisense RNA 1
ZC3H13: Zinc finger CCCH-type containing 13
